# Analysis of crystallization data in the Protein Data Bank

**DOI:** 10.1107/S2053230X15014892

**Published:** 2015-09-23

**Authors:** Jobie Kirkwood, David Hargreaves, Simon O’Keefe, Julie Wilson

**Affiliations:** a Department of Chemistry, University of York, York YO10 5DD, England; b AstraZeneca, Darwin Building, Cambridge Science Park, Cambridge CB4 0WG, England; c Department of Computer Science, University of York, York YO10 5DD, England; d Department of Mathematics, University of York, York YO10 5DD, England

**Keywords:** crystallization, pH, isoelectric point, PDB, data, statistics, database, proteins

## Abstract

The Protein Data Bank (PDB) is the largest available repository of solved protein structures and contains a wealth of information on successful crystallization. Many centres have used their own experimental data to draw conclusions about proteins and the conditions in which they crystallize. Here, data from the PDB were used to reanalyse some of these results. The most successful crystallization reagents were identified, the link between solution pH and the isoelectric point of the protein was investigated and the possibility of predicting whether a protein will crystallize was explored.

## Abstract 2

In a large-scale study using data from the Protein Data Bank, some of the many reported findings regarding the crystallization of proteins were investigated.

## 1. Introduction   

The Protein Data Bank (PDB) is an open-access online repository containing information about solved protein structures (Berman *et al.*, 2000 ▸). Along with the atomic co­ordinates, associated metadata may include the sequence of the protein, its species of origin and details of expression and structure determination. From seven structures in 1971, the number of structures in the databank has grown to over 109 000 (Protein Data Bank, 2015 ▸). The predominant method of structure determination, accounting for 89% of the entries, is X-ray crystallography. However, only ∼18% of purified proteins produce diffraction-quality crystals, with the highest attrition rate being at the crystallization stage (TargetTrack, 2015 ▸).

Various methods have been developed to predict whether a protein will crystallize based only on features derived from protein sequences (Jahandideh & Mahdavi, 2012 ▸). Features include numerical variables representing various biophysical properties, such as the isoelectric point (pI) and the grand average of hydropathy (GRAVY; Kyte & Doolittle, 1982 ▸), as well as the frequencies of dipeptide and tripeptide amino-acid compositions. Different feature sets have been calculated and used to train machine-learning algorithms, including neural networks (Kurgan *et al.*, 2009 ▸; Overton *et al.*, 2011 ▸). Here, we use a feed-forward neural network to classify protein sequences as either crystallizable or noncrystallizable and apply the trained classifier to data in the PDB.

Researchers have attempted to improve crystallization success rates by using statistical analysis of crystallization data repositories. The success rates of various crystallization reagents have been estimated (Rupp & Wang, 2004 ▸) and minimal sets of conditions that could crystallize most proteins in a given data set have been obtained (Kimber *et al.*, 2003 ▸; Page *et al.*, 2003 ▸). Scientists from structural genomics centres recently documented their support for mining data to improve crystallization, but commented that the range of proteins should be diverse (Newman, Bolton *et al.*, 2012 ▸; Gorrec, 2014 ▸). Although the PDB contains a wide variety of protein structures, the corresponding information on crystallization has been difficult to access on a large scale. Crystallization details are captured as free-text input held in the ‘REMARK 280’ lines of a PDB file and require standardiziation in order to extract useful information. For example, ammonium sulfate has been recorded as at least 30 different abbreviations and text variants (Peat *et al.*, 2005 ▸). We use a standardized snapshot of the PDB with crystallization conditions for 63 002 PDB entries that overcomes problems with inconsistencies in nomenclature (Fazio *et al.*, 2014 ▸; Newman *et al.*, 2014 ▸) to revisit some previously reported analyses. We investigate the relationship between the pI of a protein and the pH at which it crystallizes, using an estimate for the pH that takes into account the effect of other chemicals on the buffer pH, and identify the most prevalent chemicals in the PDB.

## 2. Methods and materials   

Each PDB ID in the standardized PDB (obtained from Fazio *et al.*, 2014 ▸) is associated with a protein sequence and the components of the crystallization solution. The data are not completely clean; for example, one entry has a reported concentration of 200 000 m*M* magnesium formate and in other entries a chemical appears more than once in the parsed data. After removing entries with concentrations higher than the maximum solubility levels and those with repeated chemicals, the number of PDB IDs was reduced to 60 999 to form the data set that we refer to as PDB-RAW.

Some proteins have been deposited in the PDB many times with different ligands, modifications or crystallization space groups. For example, hen egg-white lysozyme (*Gallus gallus* lysozyme) is associated with 460 X-ray structures. While redundancy is useful for some types of analysis, in other cases it skews the results. We therefore considered the data at different levels of redundancy.

**Figure 1:**
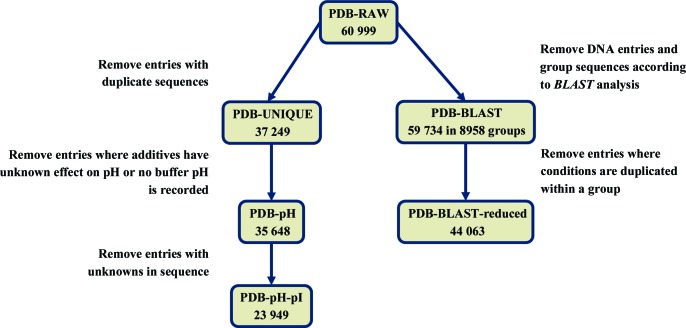
The structure of the data used for different types of analysis, showing the number of PDB entries in the various data subsets.

DNA sequences and duplicate identical protein sequences were removed from PDB-RAW to give the subset PDB-UNIQUE comprising 37 249 PDB entries. The PDB-RAW protein sequences were also analysed using *BLAST* (Madden, 2012 ▸) with a *p*-value of 10^−7^ to determine whether sequences should be considered to be the same. The PDB-BLAST subset consists of protein entries grouped according to the *BLAST* analysis, with 8958 groups each containing between one and 2115 IDs, giving 59 734 entries in total. The PDB-BLAST data set was reduced to 44 063 PDB entries by removing duplicate entries with the same experimental conditions for the same protein (but keeping all entries for the same protein where the experimental conditions differ), referred to as PDB-BLAST-reduced (Fig. 1 ▸).

### 2.1. Estimation of pH   

One of the most important parameters in crystallization is the pH of the experiment (McPherson, 1989 ▸; Newman, Sayle *et al.*, 2012 ▸). It is usually the pH of the buffer component that is recorded rather than the pH of the final crystallization solution, which can differ by up to three pH units (Bukrinsky & Poulsen, 2001 ▸; Wooh *et al.*, 2003 ▸). Kirkwood *et al.* (2015 ▸) describe the use of a neural network to provide a more accurate estimate of the pH of the crystallization solution. The network was trained to predict the effect of different chemical groups [salts, salts of weak acids, organics, polyethylene glycols (PEGs), compounds containing ammonia, hydroxide and dihydrogen salts] on the buffer pH, which can then be adjusted according to the concentrations of the other chemicals in the solution. The network cannot predict the effect of chemicals that do not belong to a group used in training and requires the buffer pH to be known. Therefore, a further 1601 entries involving chemicals for which the effect on pH is unknown or with no recorded buffer pH were removed from PDB-UNIQUE to form the subset PDB-pH, as shown in Fig. 1 ▸, and the network was used to provide the pH for this subset.

### 2.2. Prediction of crystallizability   

Overton *et al.* (2008 ▸) formed the FEAT data set using 1456 sequences obtained from TargetDB (now TargetTrack; TargetTrack, 2015 ▸; Chen *et al.*, 2004 ▸). This data set comprises 50% annotated as ‘work stopped’ (noncrystallizable) and 50% annotated as ‘diffraction quality crystal’ (crystallizable). The TEST-NEW data set, consisting of a further 1000 sequences of each class (also obtained from TargetDB), was used as an independent test set. We perform a similar analysis using the same training and test data sets, but using a feed-forward neural network with the features pI, GRAVY and counts of the amino acids Asp, Cys, Gly, His, Met, Phe, Pro, Ser, Thr, Trp and Tyr used previously in an approach using Parzen window density estimation (Overton *et al.*, 2008 ▸). We used the Levenberg–Marquardt method in *Matlab* (MathWorks) to train the network with two hidden layers, each having two nodes with sigmoid-tangent transfer functions. The trained model was then used to predict sequences found in the PDB.

## 3. Results and discussion   

**Figure 2:**
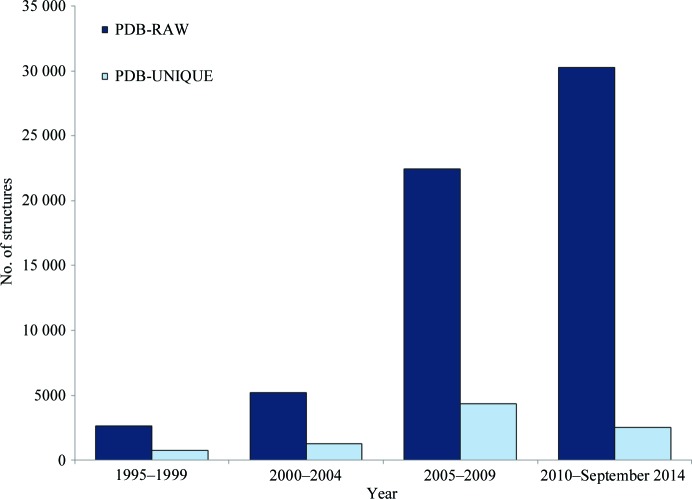
The number of structures deposited in the PDB in five-year periods since 1995. The number of structures deposited is shown together with the number of structures within each of the groups PDB-RAW and PDB-UNIQUE.

Fig. 2 ▸ shows the numbers of structures in the PDB-RAW and PDB-UNIQUE data sets plotted in five-year periods. One explanation for the decrease in the proportion of significantly different structures is the focus on drug discovery, with a limited number of target proteins and the need for protein–ligand complexes.

### 3.1. Analysis of chemical usage in the PDB   

The interactions between a protein and the various chemicals used in crystallization are complex and the number of possible combinations grows exponentially as the number of chemicals at different concentrations is increased (Rupp & Wang, 2004 ▸). Rather than explore chemical space randomly, most crystallization screens are designed rationally, making use of prior knowledge (Jancarik & Kim, 1991 ▸). It is therefore of interest to consider the most widely used conditions in the PDB and whether these have changed in recent years.

Table 1 ▸ shows the ten most prevalent chemicals in the PDB-BLAST-reduced data set, which includes some proteins more than once, provided that the experimental conditions used to generate the crystals differed. Polyethylene glycol 3350 (PEG 3350) is the most widely used chemical, followed closely by Tris buffer and ammonium sulfate. Considering all molecular-weight PEGs together (including monomethyl ethers) shows that this group of chemicals occurs in 15 910/44 063 (36%) of all conditions analysed. PEG (6000) was, to the best of our knowledge, first used to crystallize ‘alcohol oxidase’ in 1968 (Janssen & Ruelius, 1968 ▸). However, it was not until ten years later that McPherson (1976 ▸) studied the use of PEGs for crystallizing proteins and found that a screen containing PEGs of various weights and concentrations produced crystals for 13 of the 22 proteins tested, six of which had not been crystallized before. McPherson concluded that it may be the best initial trial reagent for crystallization.

**Figure 3:**
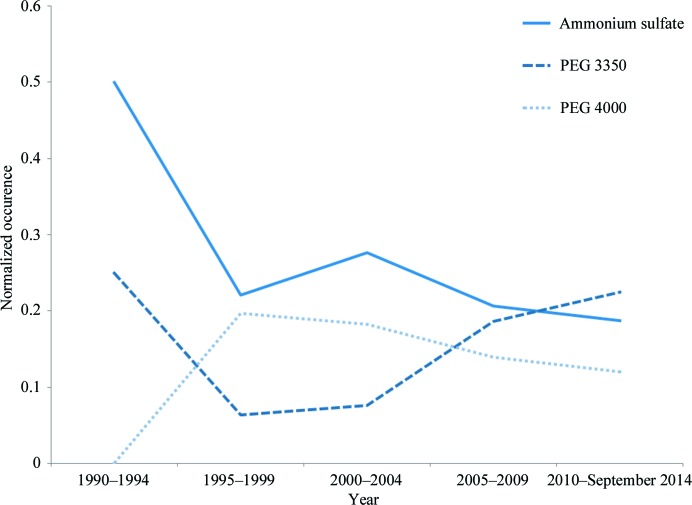
The occurrence of PEG 3350 and PEG 4000 in comparison to ammonium sulfate as found in PDB-BLAST-reduced, showing that the latter has recently been overtaken by PEG 3350 as the most prevalent chemical in crystallization trials. Normalization was performed by dividing the count of each chemical by the number of PDB entries for each five-year interval.

Subsequent studies have provided further evidence to support the use of PEGs (Hui & Edwards, 2003 ▸; McPherson, 1999 ▸). In 1984, PEG was identified as the second most widely used chemical in crystallization (Gilliland & Davies, 1984 ▸) and in 1991 PEGs were included in half (25/50) of the conditions of Jancarik and Kim’s popular sparse-matrix screen (Jancarik & Kim, 1991 ▸). PEGs are amongst the most prevalent chemicals in the PDB (Peat *et al.*, 2005 ▸), with PEG 3350 recently overtaking ammonium sulfate as the single most abundant chemical (Fig. 3 ▸).

The prevalence of PEGs has also been shown for a set of nonredundant proteins (Fazio *et al.*, 2014 ▸). The C6 metric, a similarity measure for crystallization conditions, considers PEGs with molecular weights that differ by less than a factor of two to be the same (Newman *et al.*, 2010 ▸). Together, PEG 4000 and PEG 3350 can be considered to be the most successful reagent in the history of the PDB. Although the mechanism that makes PEGs such efficacious crystallization reagents is not well understood, it seems that they may force the protein out of solution by competing with water molecules for interactions (McPherson, 1989 ▸; Lee & Lee, 1981 ▸). The varying weights and lengths enable a steric exclusion mechanism that excludes protein from areas of the solution, thereby increasing local activity and solubility (Laurent, 1963 ▸; Ward *et al.*, 1975 ▸). At neutral pH they do not require large concentrations of buffer (Kirkwood *et al.*, 2015 ▸), but they are known to degrade over time, making experiments difficult to reproduce (Ray & Puvathingal, 1985 ▸)

The other most prevalent chemicals are either buffers (HEPES, Tris and MES), which are used to control pH and are assumed to be otherwise chemically inert with respect to crystallization (although this is contestable; McPherson, 1995 ▸), or the salts ammonium sulfate, sodium and magnesium chloride and sodium acetate (also a buffer). Differential scanning fluorimetry has shown that proteins are stabilized by moderate concentrations of salt in their buffer formulations (Ristic *et al.*, 2015 ▸). Increased salt concentration may either stabilize the protein solution further (potentially allowing the protein concentration to be increased) or decrease the protein solubility causing precipitation: the so-called ‘salting-in’ and ‘salting-out’. It is assumed that the concentration of salt affects the hydration shell around the protein, which in some instances may facilitate the protein–protein interactions necessary to drive nucleation and crystallization. Zhang & Cremer (2006 ▸) showed that different ions, categorized in the Hofmeister series, vary with respect to this effect. Magnesium ions at high concentrations are able to precipitate proteins in a similar fashion to sulfate ions, although they are generally less successful in crystallization. The biological role of magnesium and calcium, both catalytically and structurally, may be reflected in the success of these salts at low concentrations in crystallization trials, with these ions often appearing in crystal structures (Kretsinger, 1976 ▸; Jayachandran *et al.*, 2007 ▸).

The salts in Table 1 ▸ have been identified previously in successful crystallization conditions using data from the PDB (Peat *et al.*, 2005 ▸) and the BMCD (Lu *et al.*, 2012 ▸). In a comparison of the success rates for 12 different salts, ammonium sulfate was identified as one of the most successful salts, although sodium malonate was found to be more than twice as successful (McPherson, 2001 ▸). Rupp & Wang (2004 ▸) also found that the success rate for ammonium sulfate was higher than the average rate for their data, whereas that for magnesium chloride was worse than average. Although these salts occur frequently in successful crystallization conditions, they are often found in combination with PEGs, making the contribution of individual components difficult to assess. In fact, 83% of solutions containing magnesium chloride also contained PEGs. Similarly, 61% of solutions containing sodium chloride and 39% of solutions containing ammonium sulfate also contained PEGs.

We found that some additives appear in very few successful crystallization solutions, with 268 chemicals used less than five times and 108 leading to a single protein structure (see Supplementary Table S1). For 83 of these 108 chemicals (76%) a protein structure was obtained for the same *BLAST* group using alternative conditions. The 25 chemicals that did lead to a unique protein structure, eight of which are ligands, might be considered a last-resort list.

### 3.2. Analysis of pH and the relationship to pI   

It is well documented that estimating the pH of a crystallization solution as that of the buffer component can be inaccurate (Kirkwood *et al.*, 2014 ▸; Newman, Sayle *et al.*, 2012 ▸; Bukrinsky & Poulsen, 2001 ▸). Chemical species such as PEGs and ammonium-containing compounds are known to degrade over time, thereby modifying the pH (Newman, Sayle *et al.*, 2012 ▸; Jurnak, 1986 ▸; Mikol *et al.*, 1989 ▸; Hampton Research, 2012 ▸). Crystallization solutions can be cooled to prevent degradation, although temperature also affects solubility (Beynon & Easterby, 1996 ▸).

**Figure 4:**
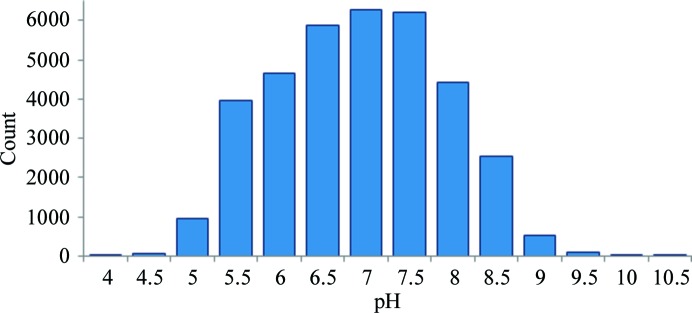
The distribution of adjusted pH values from PDB-pH obtained using a model to predict the effect on the buffer pH of other components of the crystallization solution. Values show the centre of the half-pH unit bins.

By using the method of Kirkwood *et al.* (2015 ▸) to predict the effect of nonbuffer components for crystallization solutions in the PDB-pH data set, we were able to determine an accurate distribution of pH in the PDB. For the 35 648 conditions that could be predicted, we found this to be normal with a mean close to pH 7 (Fig. 4 ▸). A normal distribution was also reported by Samudzi *et al.* (1992 ▸) in their analysis of the BMCD, but with a slightly lower mean of pH ∼6.5. Similar results were reported by Rupp & Wang (2004 ▸), but Kantardjieff & Rupp (2004 ▸) and Bonneté (2007 ▸) showed a bimodal distribution for the buffer pH with modes close to pH 6 and 9. It is interesting to note that Rupp reported two different distributions of buffer pH for crystallization solutions in the same year. A possible explanation is the source of the data, with one data set obtained from a structural genomics centre and the other from the more varied BMCD.

**Figure 5:**
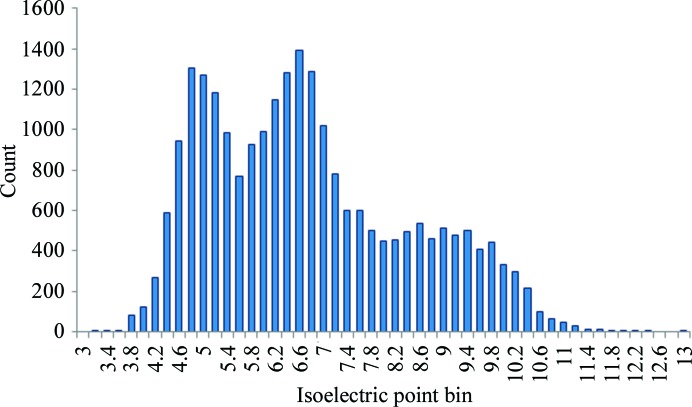
The distribution of calculated pI for 23 949 significantly different proteins in the PDB-pH-pI data set.

**Figure 6:**
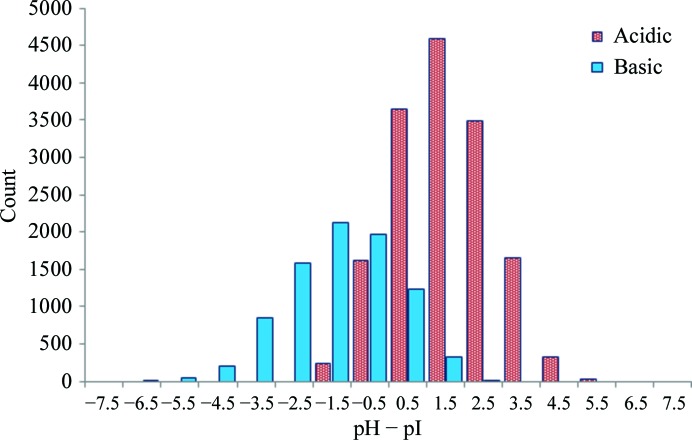
The distribution of the difference between the pH at which a structure was obtained and the isoelectric point of 23 949 proteins in the PDB-pH-pI data set. The distributions are shown separately for proteins with a pI lower than 7 (acidic) and those with a pI greater than 7 (basic). Those with a pH of precisely 7 (of which there were four) were grouped with the basic proteins.

The isoelectric point of a protein is defined as the pH at which the net charge on the protein is zero. This is a calculated parameter based on the assumption that charged residues are not buried in the hydrophobic core of the molecule and are therefore solvent-accessible. In order to concentrate a protein solution for crystallization experiments it is generally accepted that a buffer pH should be chosen taking the protein pI into consideration to avoid solubility issues (Luft *et al.*, 2011 ▸; Zhang *et al.*, 2013 ▸). It is possible to calculate the pI based on the primary sequences recorded in the PDB and to look for correlation with the experimental pH. This has been performed before and no significant correlation has been found (Page *et al.*, 2003 ▸; Huber & Kobe, 2004 ▸; Wooh *et al.*, 2003 ▸), but here we use pH values adjusted to account for the chemicals in the crystallization solution in addition to the buffer. Isoelectric points were determined for the 23 949 entries in PDB-UNIQUE for which the full sequence is known and an accurate pH can be determined (PDB-pH-pI). Previous studies suggested the pI to be bimodally distributed (Canaves *et al.*, 2004 ▸; Kantardjieff & Rupp, 2004 ▸), whereas we observe a trimodal distribution for the PDB data with peaks close to pH 4.8, 6.6 and 9.0 (Fig. 5 ▸). The relationship between the pI of proteins and the pH at which they have been crystallized is shown in Fig. 6 ▸. Acidic proteins, *i.e.* those with a pI below 7, tend to crystallize about one pH unit above their pI, whereas basic proteins tend to crystallize below their pI by around 1.5–3 pH units. These results support previous findings (Kantardjieff *et al.*, 2004 ▸; Kantardjieff & Rupp, 2004 ▸; Charles *et al.*, 2006 ▸).

### 3.3. Analysis of protein properties   

**Figure 7:**
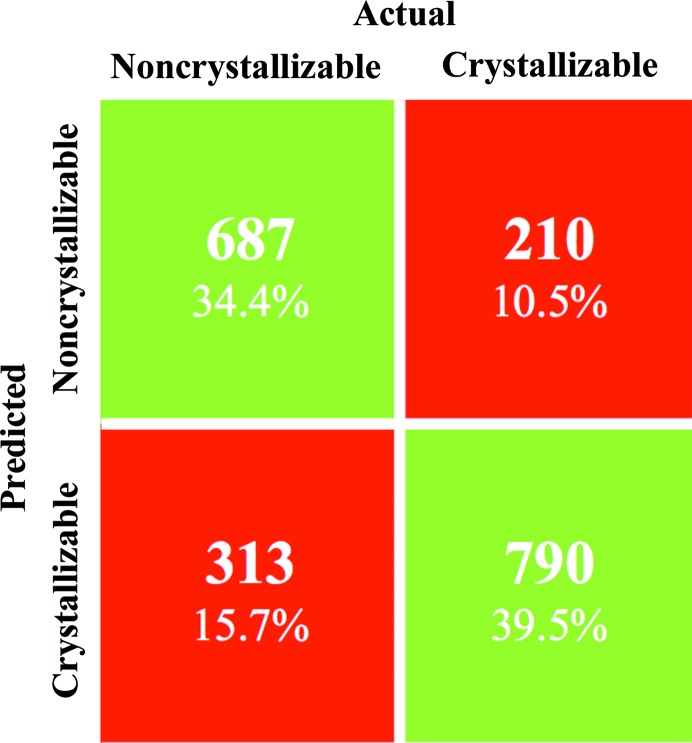
Confusion matrix showing the results for the sequence-based prediction of crystallizability: those sequences considered to be crystallizable (‘diffraction quality crystal’) and those considered noncrystallizable (‘work stopped’). The results on the 2000 sequences in the independent test set show that 1477 (73.9%) can be predicted correctly, with the correctly predicted noncrystallizable and crystallizable sequences accounting for 687/2000 (34.4%) and 790/2000 (39.5%), respectively. Thus, 210/2000 (10.5%) of crystallizable and 313/2000 (15.7%) of noncrystallizable sequences were incorrectly predicted.

Fig. 6 ▸ shows that the majority of crystallized proteins are acidic. In general, proteins that are both acidic and hydrophilic are considered to be more likely to crystallize (Canaves *et al.*, 2004 ▸), whilst those that are both basic and hydrophobic are less likely. As both the acidity and the hydrophobicity can be calculated from a protein sequence, a prediction can be made as to whether a protein will crystallize (Smialowski *et al.*, 2006 ▸; Overton & Barton, 2006 ▸; Slabinski *et al.*, 2007 ▸; Overton *et al.*, 2008 ▸, 2011 ▸; Mizianty & Kurgan, 2009 ▸; Kurgan *et al.*, 2009 ▸; Babnigg & Joachimiak, 2010 ▸; Kandaswamy *et al.*, 2010 ▸). The confusion matrix in Fig. 7 ▸ shows that 73.9% of the sequences in the TEST-NEW data set were predicted correctly by our neural network, with slightly more true positives (790) than true negatives (687). However, this model does not generalize to the PDB data, with only 55% of sequences correctly predicted as crystallizable. Restricting the PDB data to sequences submitted between July 2006 and December 2008 to reflect the TEST-NEW data set did little to improve the accuracy, with just 58% (3180/5453) correctly predicted. As shorter sequences are not well represented in the FEAT data set, we also tried restricting the PDB data to sequences with more than 99 amino acids, resulting in an accuracy of just 58% (13 233/22 829). To be sure that the low prediction rates were not particular to our network, we used the online predictor *CRYSTALP*2 (Kurgan *et al.*, 2009 ▸) with a random sample of 1000 sequences from the PDB with between 100 and 1000 residues. Again the accuracy was low, with just 60% of the sequences classified as ‘crystallizable’.

In the original training and test data sets, crystallizable proteins were obtained from TargetDB (TargetTrack) if annotated as having ‘diffraction quality crystals’, but specifically not ‘in PDB’ in the ‘status’ field (Kurgan *et al.*, 2009 ▸). The motivation for excluding sequences resulting in PDB structures is not given. It seems there are sequence differences between proteins designated as producing diffraction-quality crystals in TargetDB and those that result in a structure deposited in the PDB. One possible explanation is the fact that only structural genomics targets are included in TargetDB and may be restricted, for example owing to particular medical interests, whereas structures deposited in the PDB are from a wider, and potentially more difficult to crystallize, range of proteins. We cannot assume that diffraction data were actually collected for proteins annotated as producing diffraction-quality crystals; in fact, diffraction data are collected for about a third of the structural genomics targets for which crystals are obtained, and only two-thirds of these result in a protein structure in the PDB (Westbrook *et al.*, 2003 ▸).

## 4. Conclusions   

Statistical analysis of the data from successful experiments can provide useful information for the development of new crystallization strategies. Our analysis of the PDB broadly confirms previous findings, with the distribution of pH values as expected, and justifies the use of PEG as the ‘go to’ reagent of choice and shows magnesium chloride to be a successful crystallization agent, albeit predominantly in solution with PEG. The lack of correlation between the pH of crystallization and pI was confirmed and the patterns observed, with acidic proteins tending to crystallize at a pH just above their pI and basic proteins tending to crystallize below their pI, can be attributed to the fact that, on average, proteins crystallize at neutral pH. Sequence-based algorithms to predict the propensity of a protein to crystallize (Smialowski *et al.*, 2006 ▸; Jahandideh & Mahdavi, 2012 ▸) have been optimized using targets from particular protein families and do not appear to generalize to proteins with structures deposited in the PDB.

In order to retrain classification algorithms, suitably unbiased data on unsuccessful crystallization trials would also be needed. Such data are also necessary to investigate the relationship between protein properties and the conditions that result in crystals (Hennessy *et al.*, 2000 ▸). This could potentially allow properties that can be measured or calculated before crystallization trials begin to be used to predict the best initial conditions to try.

The standardized PDB facilitates data-mining studies and could be used to investigate other indicators of the ability of a protein to crystallize including, for example, molecular weight and domain structure. Is low molecular weight better than high molecular weight, are single-domain proteins more likely to crystallize than multi-domain proteins and is an oligomeric state multimer better than a monomer? However, consistency in the reporting of metadata is crucial to such studies and the use of IUPAC names for all chemical entries in the PDB (not just ligands) would certainly help.

## Supplementary Material

Supplementary Table S1.. DOI: 10.1107/S2053230X15014892/nj5235sup1.pdf

## Figures and Tables

**Table 1:** The ten most prevalent chemical species with the number of entries in the PDB-BLAST-reduced data set consisting of 44063 PDB entries

Rank	Chemical	Count
1	Polyethylene glycol 3350	9264
2	Tris	8375
3	Ammonium sulfate	8225
4	HEPES	5795
5	Polyethylene glycol 4000	5637
6	Sodium chloride	5248
7	Sodium acetate	5194
8	Polyethylene glycol 8000	4095
9	Magnesium chloride	3845
10	MES	3664
