# Supplementary material for: Analysis of crystallization data in the Protein Data Bank
Source: Acta Crystallogr F Struct Biol Commun. 2015 Sep 23;71(Pt 10):1228–34. doi: 10.1107/S2053230X15014892 (PMC4601584; doi:10.1107/S2053230X15014892)
Supplement: Supplementary file 1 [file f-71-01228-sup1.pdf]

STRUCTURAL BIOLOGY COMMUNICATIONS

Volume 71 (2015)

Supporting information for article:

Analysis of crystallization data in the Protein Data Bank

Jobie Kirkwood, David Hargreaves, Simon O'Keefe and Julie Wilson

Table S1 Supplementary Table 1A list of 108 chemicals found only once in the PDB

A total of 25 chemicals are found in crystallization solutions that uniquely crystallise a protein. * indicates that this chemical is in a solution that crystallizes only one protein.

Chemical
1,2-CYCLOHEXANEDICARBOXYLIC ACID *
1,4-CYCLOHEXANEDICARBOXYLIC ACID *
1,5-HEXANEDIOL
1-METHYLIMIDAZOLIUM FORMATE
2,5-PYRIDINEDICARBOXYLIC ACID *
2,6-DIMETHYL-4-HEPTYL-BETA-D-MALTOPYRANOSIDE
2'-DEOXYADENOSINE
2-PHOSPHOGLYCOLIC ACID
3-PENTANONE
4-AMINOBENZOIC ACID
4-AMINOBUTANOIC ACID
ACETAMIDE
ACETATE-ADA-BICINE
ACETYLSALICYLIC ACID *
ADENOSINE DIPHOSPHATEGLUCOSE *
AMMONIUM MALATE
ANAPOE-X-114
ARABINOSE-5-PHOSPHATE
AZELAIC ACID
BARIUM ACETATE *
BENZOIC ACID
BENZYLDODECYLDIMETHYLAMMONIUM BROMIDE
BETA-ALANINE
BETA-CYCLODEXTRIN

BIS TRIS HEPES

C10E5

C8E6

CALCIUM BROMIDE

CALCIUM SULFATE *

CHOLINE CHLORIDE

CITRATE-HEPES-CHES

CITRULLINE

COBALT(II) ACETATE

COENZYME B12

CYCLOHEXYLETHANOYL-N-HYDROXYETHYLGLUCAMIDE *

CYSTINE

DEOXYCYTIDINE MONOPHOSPHATE

DIHYDROQUERCETIN

DIISOPROPYL FLUOROPHOSPHATE

DL-XYLOSE

DODECYLTRIMETHYLAMMONIUM CHLORIDE

D-RIBOSE

ECTOINE

ETHIDIUM BROMIDE *

ETHYLENEDIAMINE DIHYDROCHLORIDE

EUROPIUM NITRATE *

FOS-CHOLINE-12

FOS-CHOLINE-9 *

GLUTATHIONE, OXIDISED

GLYCERALDEHYDE

GLYCEROL 3-PHOSPHATE

GLYCEROL ETHOXYLATE

GLY-PHE

GLY-TYR

GUANOSINE

HEGA-9 *

HEXADECANEDIOIC ACID

HEXADECYLTRIMETHYLAMMONIUM CHLORIDE

HEXAETHYLENE GLYCOL DECYL ETHER *

HISTAMINE

HOMOSERINE

JEFFAMINE D-2000 *

JEFFAMINE M-2070

JEFFAMINE SD-2001 *

LANTHANUM CHLORIDE

LEU-GLY-GLY

LITHIUM CACODYLATE

LUTETIUM ACETATE *

MAGNESIUM CALCIUM SULFATE

MEGA 9

MELIBIOSE *

MERCURY(II) ACETATE

MERCURY(II) SULFATE *

MERPOL HCS

N,N-DIMETHYLHEXYLAMINE-N-OXIDE *

N-ACETYLMURAMIC ACID

NICKEL ACETATE

N-NONYL-B-D-THIOMALTOSIDE

ORNITHINE

PHENYLUREA

POTASSIUM MOPS *

PROTEASE

RUBIDIUM CACODYLATE

SALICYLAMIDE *

SAMARIUM ACETATE

SAMARIUM NITRATE

SEBACIC ACID

SODIUM CHLORITE

SODIUM CITRATE-POTASSIUM CITRATE

SODIUM DIHYDROGEN PHOSPHATE-DISODIUM HYDROGEN PHOSPHATE

SODIUM DODECANOYL SARCOSINE *

SODIUM SELENATE

STACHYOSE

SUBERIC ACID

SUCROSE MONOLAURATE

TERGITOL NP-40

TETRAETHYLENE GLYCOL

TETRAHYDROFOLIC ACID

THIMEROSAL

THULIUM CHLORIDE

THYMOL

TRANS-ACONITIC ACID *

TRIS AMPD

TRISODIUM CITRATE-CITRIC ACID

TRISODIUM EDTA

VANILLIN *

ZWITTERGENT 3-08 *

ZWITTERGENT 3-16
